# Association of early oxygen therapy parameters with retinopathy of prematurity stages in preterm infants with respiratory distress syndrome: a gestational age-stratified analysis

**DOI:** 10.3389/fped.2026.1911394

**Published:** 2026-08-12

**Authors:** Shanshan Chen, Rong Li, Na Shi, Qinxing Xie, Wenqiang Liu

**Affiliations:** Department of Pediatrics, The Affiliated Hospital of Xuzhou Medical University, Xuzhou, Jiangsu, China

**Keywords:** early oxygen therapy parameters, gestational age stratification, preterm infant, respiratory distress syndrome, retinopathy of prematurity stage

## Abstract

**Objective:**

To investigate the associations between early oxygen-related parameters and ROP severity in preterm infants with respiratory distress syndrome (RDS), and to evaluate their discriminative performance for severe ROP.

**Methods:**

This retrospective study included 825 preterm infants (24–<37 weeks) with RDS stratified by gestational age (32–<37, 28–<32, and 24–<28 weeks). Early oxygen parameters during the first 7 days after birth included cumulative oxygen duration, mean and maximum FiO_2_, SpO_2_ target range time proportion (91%–95%), and intermittent hypoxia frequency. Correlation analysis, multivariable logistic regression, and ROC analyses were performed.

**Results:**

ROP incidence was 12.97% (107/825) and increased with decreasing gestational age (1.21%, 25.80%, and 59.57%, respectively; *P* < 0.001). Compared with mild ROP, severe ROP showed longer oxygen duration, higher FiO_2_ exposure, more intermittent hypoxia episodes, and lower SpO_2_ target range time proportion (all *P* < 0.05). After adjustment for gestational age, birth weight, and surfactant use, none of the oxygen-related parameters remained independently associated with severe ROP. ROC analysis among infants with ROP showed moderate discrimination for individual oxygen parameters (AUC: 0.705–0.761), whereas models incorporating oxygen parameters showed limited improvement beyond gestational age and birth weight.

**Conclusion:**

Early oxygen-related parameters were associated with ROP severity but showed limited independent discriminative value beyond gestational age and birth weight. They may represent candidate markers for risk characterization, although prospective validation is required before clinical application.

## 1 Introduction

Retinopathy of prematurity (ROP) is a common and severe retinal vasoproliferative disorder in preterm infants and a major cause of avoidable childhood blindness (1–3). Advances in perinatal medicine and neonatal intensive care have significantly improved survival rates for very low birth weight and extremely preterm infants, with a continued increase in the number of ROP cases (4). In developing countries especially, variations in oxygen therapy management and neonatal care capacity across regions have led to marked changes in the ROP disease spectrum and severity. Previous studies have identified lower gestational age, low birth weight, prolonged mechanical ventilation, and increased oxygen exposure as important risk factors for ROP (5). However, the methods for evaluating oxygen therapy parameters vary across studies, leading to ongoing debate regarding the relationship between oxygen management and ROP.

The pathogenesis of ROP is closely linked to abnormal retinal vascular development. Normally, fetal retinal vessels gradually grow toward the periphery during the second and third trimesters. Preterm infants, being removed prematurely from the relatively hypoxic intrauterine environment, are exposed to a relatively hyperoxic state after birth, which suppresses vascular endothelial growth factor (VEGF) expression and arrests retinal vascular growth. Subsequently, as metabolic demands increase and local hypoxia develops, VEGF becomes abnormally elevated again, inducing pathological neovascularization and ultimately leading to ROP development and progression (6, 7). Thus, oxygen therapy is both an essential supportive measure for preterm infant survival and a key environmental factor in ROP development. In particular, preterm infants with respiratory distress syndrome (RDS) often require prolonged oxygen therapy or even mechanical ventilation due to lung immaturity, surfactant deficiency, and impaired oxygenation. Their oxygen exposure levels are generally much higher than those of preterm infants without RDS, making them more susceptible to ROP-related complications (8).

In recent years, with the emergence of precision oxygen therapy concepts, it has been clinically recognized that ROP development relates not only to whether oxygen is given but also to the dynamic characteristics of oxygen exposure. Traditional studies have focused largely on a single fraction of inspired oxygen (FiO_2_) level or total oxygen duration (9), with relatively less attention given to dynamic parameters such as mean FiO_2_, maximum FiO_2_, time proportion of pulse oxygen saturation (SpO_2_) within target range, and intermittent hypoxic events. Existing evidence suggests that oxygen fluctuations may induce oxidative stress and retinal vascular damage more readily than sustained hyperoxia (10). Frequent hypoxia-reoxygenation cycles can generate large amounts of reactive oxygen species, triggering inflammatory responses and abnormal vascular proliferation. Moreover, insufficient time maintaining SpO_2_ within the target range may indicate poor stability of oxygen therapy, thereby further increasing ROP risk (11). Therefore, relying solely on traditional oxygen duration to assess ROP risk may be insufficient, and constructing a more comprehensive oxygen exposure evaluation system is important for early ROP identification and risk stratification.

Several limitations exist in current studies on the relationship between oxygen parameters and ROP severity. First, most studies include only very low birth weight or very preterm infants, lacking systematic comparisons across different gestational age strata. Second, some studies focus mainly on whether ROP occurs, with less analysis of ROP stages and the risk of severe ROP. Third, there are few reports on dynamic oxygen parameters such as intermittent hypoxic events and proportion of time within SpO_2_ target range, and there is no uniform clinical standard for their evaluation. Additionally, preterm infants of different gestational ages show marked differences in lung maturity, oxygen requirements, and retinal vascular development status, and the effect of oxygen exposure on ROP may be gestational age-dependent. Accordingly, it is necessary to further explore the relationship between oxygen parameters and ROP severity based on gestational age stratification.

Given this background, this retrospective study stratified 825 preterm infants with RDS by gestational age to compare ROP incidence, stage characteristics, and early oxygen parameters across strata. Correlation and receiver operating characteristic (ROC) curve analyses were used to evaluate the discriminative value of oxygen parameters for severe ROP. The goal was to explore clinical patterns of ROP development from a dynamic oxygen exposure perspective and to provide evidence for optimizing oxygen therapy and early risk warning in this population.

## 2 Materials and methods

### 2.1 Patient data

A retrospective cohort study design was used. A total of 825 preterm infants with RDS who met the inclusion criteria and were hospitalized in the Department of Neonatology of our hospital from January 2022 to December 2025 were enrolled. According to the World Health Organization (WHO) subclassification of preterm birth (https://www.who.int/news-room/fact-sheets/detail/preterm-birth), gestational age was divided into three strata: 32–<37 weeks, 28–<32 weeks, and 24–<28 weeks. The inclusion criteria were: (1) gestational age 24–<37 weeks; (2) meeting the clinical and chest radiographic diagnostic criteria for neonatal RDS according to the 2019 European consensus guidelines on the management of neonatal RDS (12); (3) admitted within 24 h after birth and receiving oxygen therapy; (4) undergoing at least one standardized ROP screening in our hospital and followed up until complete retinal vascularization or ROP regression; (5) complete clinical data, including blood gas analysis, oxygen therapy records, and ROP screening reports. The exclusion criteria included: (1) complicated with severe congenital malformations, chromosomal abnormalities or genetic defects, and inherited metabolic diseases; (2) death during hospitalization, treatment withdrawal, or discharge against medical advice; (3) congenital retinal developmental abnormalities, congenital cataracts, or other ocular structural abnormalities; (4) exogenous oxygen therapy or pulmonary surfactant (PS) treatment before admission; (5) maternal diseases during pregnancy that might independently affect ROP, such as diabetic retinopathy.

### 2.2 Collection of clinical characteristics

The following clinical data were collected from the hospital's electronic medical record system (EMRS) and nursing records: sex, gestational age, birth weight, Apgar scores (1, 5 min), antenatal corticosteroid use rate, and PS use rate (administration of corticosteroids for fetal lung maturation at any time within 24 h–7 d before delivery).

### 2.3 Collection of early oxygen therapy parameters

In this study, “early” oxygen exposure was defined as the first 7 days after birth, a period when immature retinal vessels are particularly vulnerable to oxygen-induced vaso-obliteration and oxidative stress. Historical oxygen-related data, including FiO_2_ and SpO_2_ records, were extracted from electronic medical records and nursing documentation. Two trained researchers independently reviewed and cross-checked the data.

1. Cumulative oxygen duration was defined as the total duration of oxygen therapy during the first 7 days after birth, including high-flow nasal cannula, non-invasive positive pressure ventilation, and invasive mechanical ventilation, calculated from documented start and end times.
2. Mean FiO_2_ was calculated as the arithmetic mean of all recorded FiO_2_ measurements during the first 7 days after birth. FiO_2_ values were obtained from routinely recorded oxygen therapy data, and the arithmetic mean was used to represent overall oxygen exposure during the observation period.
3. Time proportion of SpO_2_ within the target range was calculated as the percentage of valid monitoring time with SpO_2_ maintained within 91%–95% according to previous recommendations (13).
4. Intermittent hypoxia episode frequency was calculated as the average number of episodes per day. Episodes were primarily identified from continuous SpO_2_ monitoring records and defined as a decrease in SpO_2_ to <80% lasting ≥10 s followed by recovery to baseline, according to previous studies (14, 15). Nursing records were used only to confirm clinically significant events and exclude artifacts, rather than as the primary source of episode identification. Episodes related to sensor displacement or technical artifacts were excluded. Missing data were handled using linear interpolation, and cases with consecutive missing values ≥3 h were excluded.

### 2.4 Collection of ROP characteristics

ROP screening was performed by ophthalmologists at our hospital using the SUOER Wide-Field Pediatric Retinal Imaging System (Tianjin Suwei Electronic Technology Co., Ltd., SW-8000). The timing of the first screening and follow-up frequency were based on the *Guidelines for Oxygen Therapy and Prevention of Retinopathy of Prematurity in Premature Infants* issued by the Ministry of Health (16): all preterm infants with birth weight ≤2,000 g or gestational age ≤34 weeks were required to undergo screening. For infants with birth weight >2,000 g and gestational age >34 weeks but who had risk factors such as severe systemic illness or prolonged oxygen exposure, screening could be considered on a case-by-case basis. The first examination was performed at 4 weeks after birth or at a postmenstrual age of 31–32 weeks, whichever was later. Follow-up intervals were determined based on the initial ROP stage, zone, and the presence of plus disease, with repeat examinations typically conducted every 1–2 weeks until complete retinal vascularization or ROP regression/stabilization. ROP zone, stage, and plus disease were assessed according to the *International Classification of Retinopathy of Prematurity* (revised) (ICROP, 2005) and the 3rd edition of ICROP (2021) (17, 18). (1) Zones: Zone I is a circle centered on the optic disc with a radius equal to twice the distance from the disc to the fovea; Zone II extends from the edge of Zone I to the nasal ora serrata; Zone III is the remaining temporal retina. If zones differed between eyes, the more severe side was recorded. (2) Stages: Stage 1 is a demarcation line within the retina; Stage 2 is a ridge; Stage 3 is a ridge with extraretinal fibrovascular proliferation; Stage 4 is subtotal retinal detachment (Stage 4A not involving the macula, Stage 4B involving the macula); Stage 5 is total retinal detachment. If stages differed between eyes, the higher stage was recorded. (3) Plus disease was defined as present or absent, i.e., obvious dilation and tortuosity of the posterior retinal vessels (posterior plus disease) or iris neovascularization (anterior plus disease). (4) Treatment group assignment followed the ETROP trial criteria (19): Infants who met treatment threshold (any stage ROP in Zone I with plus disease, or Stage 3 ROP in Zone I with or without plus disease, or Stage 2 or 3 ROP in Zone II with plus disease, or aggressive posterior ROP) were assigned to the severe ROP group and required anti-VEGF intravitreal injection, retinal laser photocoagulation, or vitreoretinal surgery; Stage 4 and 5 ROP were also assigned to the severe ROP group. Those who did not meet treatment threshold and had spontaneous regression or required only periodic observation were assigned to the mild ROP group.

### 2.5 Statistical methods

SPSS 27.0 was used for statistical analysis. Continuous variables were tested for normality using the Shapiro–Wilk method and expressed as mean ± standard deviation or median (interquartile range), as appropriate. Comparisons between groups were performed using independent-samples *t*-test, Welch *t*-test, Mann–Whitney *U* test, ANOVA, or Kruskal–Wallis test as appropriate. Categorical variables were compared using the *χ*^2^ test or Fisher's exact test. Spearman correlation analysis was used to assess the association between early oxygen parameters and ROP stage. Among infants with ROP, multivariable logistic regression analyses were performed to evaluate adjusted associations between oxygen parameters and severe ROP. Gestational age, birth weight, and surfactant use were included as adjustment variables, and each oxygen parameter was entered separately to reduce potential multicollinearity. Variance inflation factor (VIF) analysis was performed, with VIF <5 considered acceptable. ROC curve analyses were performed among infants with ROP to evaluate the discriminative performance of oxygen parameters and logistic regression models for distinguishing severe ROP from mild ROP. The ROC curves were generated using the predicted probabilities derived from logistic regression models. The area under the curve (AUC), sensitivity, specificity, and Youden index were calculated. To evaluate whether oxygen parameters provided additional discriminative value beyond baseline maturity-related factors, models based on gestational age and birth weight were compared with models incorporating individual or combined oxygen parameters. A two-sided *P* value <0.05 was considered statistically significant.

## 3 Results

### 3.1 Comparison of RDS and ROP incidence across gestational age strata

Among the 825 preterm infants with RDS, the gestational age stratification was as follows: 495 cases (60.00%) in the 32–<37 weeks group, 283 (34.30%) in the 28–<32 weeks group, and 47 (5.70%) in the 24–<28 weeks group. The overall ROP incidence was 12.97% (107/825). ROP incidence increased significantly with decreasing gestational age: 1.21% (6/495) in the 32–<37 weeks group, 25.80% (73/283) in the 28–<32 weeks group, and 59.57% (28/47) in the 24–<28 weeks group, with a statistically significant difference among the three groups (*P* < 0.05). Pairwise comparisons showed that ROP incidence in both the 28–<32 weeks group and the 24–<28 weeks group was significantly higher than that in the 32–<37 weeks group, and the 24–<28 weeks group also had a significantly higher incidence than the 28–<32 weeks group (*P* < 0.05) (Table 1).

**Table 1:** Comparison of RDS and ROP incidence across gestational age strata in preterm infants.

Condition	Total (*n* = 825)	32–<37 w (*n* = 495)	28–<32 w (*n* = 283)	24–<28 w (*n* = 47)	*χ* ^2^	*P*
RDS	825 (100.00)	495 (60.00)	283 (34.30)	47 (5.70)	—	—
ROP	107 (12.97)	6 (1.21)	73 (25.80)^a^	28 (59.57)^a^^,^^b^	192.304	<0.001

RDS, respiratory distress syndrome; ROP, retinopathy of prematurity. Compared with 32–<37 weeks; compared with 28–<32 weeks.

a *P* < 0.05.

b *P* < 0.05.

### 3.2 Comparison of clinical characteristics and early oxygen parameters across gestational age strata in preterm infants with RDS

Sex distribution among the three groups showed no statistically significant difference (*P* > 0.05). With decreasing gestational age, gestational age, birth weight, 1-min and 5-min Apgar scores all showed a decreasing trend, while antenatal corticosteroid use rate and PS use rate showed increasing trends, with statistically significant differences among groups (*P* < 0.05). Regarding early oxygen parameters, with decreasing gestational age, cumulative oxygen duration, mean FiO_2_, maximum FiO_2_, and intermittent hypoxia episode frequency each increased sequentially and significantly (*P* < 0.05), while time proportion of SpO_2_ within target range decreased sequentially and significantly (*P* < 0.05). Except for antenatal corticosteroid use rate between the 24–<28 weeks group and the 28–<32 weeks group (no significant difference), all pairwise comparisons for the other indicators showed statistically significant differences (*P* < 0.05) (Table 2).

**Table 2:** Comparison of clinical characteristics and early oxygen parameters across gestational age strata in preterm infants with RDS (*n* = 825).

Characteristic/Parameter	32–<37 w (*n* = 495)	28–<32 w (*n* = 283)	24–<28 w (*n* = 47)	*F/χ* ^2^	*P*
Sex (*n*)				0.425	0.809
Male	268 (54.14)	149 (52.65)	27 (57.45)	—	—
Female	227 (45.86)	134 (47.35)	20 (42.55)	—	—
Gestational age (weeks)	34.38 ± 1.19	29.71 ± 1.03^a^	26.02 ± 0.90^a^^,^^b^	2,704.314	<0.001
Birth weight (kg)	2.42 ± 0.35	1.46 ± 0.29^a^	0.89 ± 0.15^a^^,^^b^	1,807.882	<0.001
1-min Apgar (score)	7.76 ± 1.28	6.46 ± 1.59^a^	5.26 ± 1.66^a^^,^^b^	106.967	<0.001
5-min Apgar (score)	8.66 ± 0.93	7.88 ± 1.25^a^	7.13 ± 1.36^a^^,^^b^	63.721	<0.001
Antenatal corticosteroid use (%)	265 (53.54)	210 (74.20)^a^	37 (78.72)^a^	38.549	<0.001
PS use (%)	138 (27.88)	201 (71.02)^a^	42 (89.36)^a^^,^^b^	172.251	<0.001
Cumulative oxygen duration (h)	102.70 ± 17.93	140.67 ± 23.82^a^	165.49 ± 5.25^a^^,^^b^	1,597.130	<0.001
Mean FiO_2_ (%)	27.64 ± 4.19	33.90 ± 6.67^a^	41.79 ± 9.12^a^^,^^b^	27.64 ± 4.19	<0.001
Maximum FiO_2_ (%)	42.87 ± 11.42	59.04 ± 16.84^a^	75.55 ± 16.11^a^^,^^b^	42.87 ± 11.42	<0.001
SpO_2_ target range time proportion (%)	91.07 ± 8.28	80.63 ± 13.11^a^	68.53 ± 14.73^a^^,^^b^	117.252	<0.001
Intermittent hypoxia frequency (episodes/d)	3.33 ± 2.06	8.67 ± 4.48^a^	16.04 ± 6.88^a^^,^^b^	250.900	<0.001

Compared with 32–<37 weeks; compared with 28–<32 weeks. RDS, respiratory distress syndrome; PS, pulmonary surfactant; FiO_2_, fraction of inspired oxygen; SpO_2_, pulse oxygen saturation.

a *P* < 0.05.

b *P* < 0.05

### 3.3 Comparison of clinical characteristics and early oxygen parameters across gestational age strata in preterm infants with ROP

Among the 107 infants who developed ROP, sex distribution and antenatal corticosteroid use rate showed no statistically significant differences among the three groups (*P* > 0.05). Gestational age, birth weight, and 1-min and 5-min Apgar scores all decreased significantly with decreasing gestational age (*P* < 0.05). PS use rate was significantly higher in both the 28–<32 weeks and 24–<28 weeks groups compared with the 32–<37 weeks group (*P* < 0.05). Regarding early oxygen parameters, cumulative oxygen duration, mean FiO_2_, maximum FiO_2_, and intermittent hypoxia episode frequency each increased sequentially with decreasing gestational age, while SpO_2_ target range time proportion decreased sequentially; differences among the three groups were statistically significant for all parameters (*P* < 0.05), and pairwise comparisons between any two groups were also statistically significant (*P* < 0.05) (Table 3).

**Table 3:** Comparison of clinical characteristics and early oxygen parameters across gestational age strata in preterm infants with ROP (*n* = 107).

Characteristic/Parameter	32–<37 w (*n* = 6)	28–<32 w (*n* = 73)	24–<28 w (*n* = 28)	*F/χ* ^2^	*P*
Sex (n)				0.400	0.894
Male	3 (50.00)	41 (56.16)	17 (60.71)	—	—
Female	3 (50.00)	32 (43.84)	11 (39.29)	—	—
Gestational age (weeks)	33.83 ± 1.17	29.29 ± 0.96^a^	25.54 ± 0.79^a^^,^^b^	264.478	<0.001
Birth weight (kg)	2.39 ± 0.46	1.34 ± 0.27^a^	0.81 ± 0.13^a^^,^^b^	109.516	<0.001
1-min Apgar (score)	7.50 ± 1.23	5.82 ± 1.61^a^	4.64 ± 1.52^a^^,^^b^	10.314	<0.001
5-min Apgar (score)	8.67 ± 0.52	7.34 ± 1.25^a^	6.64 ± 1.28^a^^,^^b^	7.546	0.008
Antenatal corticosteroid use (%)	3 (50.00)	52 (71.23)	21 (75.00)	1.599	0.463
PS use (%)	2 (33.33)	56 (76.71)^a^	26 (92.86)^a^	9.670	0.006
Cumulative oxygen duration (h)	125.17 ± 19.74	155.59 ± 17.37^a^	166.21 ± 3.92^a^^,^^b^	22.967	<0.001
Mean FiO_2_ (%)	30.17 ± 3.82	38.05 ± 5.78^a^	46.36 ± 7.53^a^^,^^b^	25.695	<0.001
Maximum FiO_2_ (%)	55.00 ± 11.33	69.49 ± 13.97^a^	84.00 ± 10.73^a^^,^^b^	18.111	<0.001
SpO_2_ target range time proportion (%)	86.00 ± 8.76	71.55 ± 10.00^a^	60.57 ± 11.23^a^^,^^b^	19.737	<0.001
Intermittent hypoxia frequency (episodes/d)	5.67 ± 0.52	11.55 ± 4.00^a^	18.07 ± 6.91^a^^,^^b^	101.726	<0.001

Compared with 32–<37 weeks; compared with 28–<32 weeks. ROP, retinopathy of prematurity; PS, pulmonary surfactant; FiO_2_, fraction of inspired oxygen; SpO_2_, pulse oxygen saturation.

a *P* < 0.05.

b *P* < 0.05

### 3.4 Comparison of ROP characteristics across gestational age strata

The distribution of ROP zones among the three groups of infants with ROP showed no statistically significant difference (*P* > 0.05). The distribution of ROP stages differed significantly among groups (*P* < 0.05), with the 24–<28 weeks group showing significantly higher stages than the other two groups (*P* < 0.05). The incidence of plus disease increased with decreasing gestational age, with a statistically significant difference (*P* < 0.05), and the 24–<28 weeks group had a significantly higher incidence than the other two groups (*P* < 0.05). Regarding treatment group assignment, all infants in the 32–<37 weeks group had mild ROP (100.00%); in the 28–<32 weeks group, 80.82% were mild and 19.18% severe; in the 24–<28 weeks group, severe ROP predominated (75.00%). The difference among the three groups was statistically significant (*P* < 0.05), and the proportion of severe ROP in the 24–<28 weeks group was significantly higher than that in the other two groups (*P* < 0.05) (Table 4).

**Table 4:** Comparison of ROP characteristics across gestational age strata (*n* = 107).

ROP characteristic	32–<37 w (*n* = 6)	28–<32 w (*n* = 73)	24–<28 w (*n* = 28)	*χ* ^2^ */H*	*P*
Zone				8.378	0.060
I	0 (0.00)	7 (9.59)	8 (28.57)	—	—
II	2 (33.33)	39 (53.42)	14 (50.00)	—	—
III	4 (66.67)	27 (36.99)	6 (21.43)	—	—
Stage				14.060	0.003
1	3 (50.00)	20 (27.40)	3 (10.71)	—	—
2	3 (50.00)	31 (42.47)	7 (25.00)	—	—
3	0 (0.00)	22 (30.14)	18 (64.29)	—	—
4	0 (0.00)	0 (0.00)	0 (0.00)	—	—
5	0 (0.00)	0 (0.00)	0 (0.00)	—	—
Plus disease	0 (0.00)	10 (13.70)	11 (39.29)^a^^,^^b^	8.497	0.011
Treatment group				29.822	<0.001
Mild	6 (100.00)	59 (80.82)	7 (25.00)	—	—
Severe	0 (0.00)	14 (19.18)	21 (75.00)^a^^,^^b^	—	—

Compared with 32–<37 weeks; compared with 28–<32 weeks. ROP, retinopathy of prematurity.

a *P* < 0.05.

b *P* < 0.05

### 3.5 Comparison of early oxygen parameters between different ROP severity groups

The severe ROP group showed higher cumulative oxygen duration, mean FiO_2_, maximum FiO_2_, and intermittent hypoxia episode frequency, together with a lower SpO_2_ target range time proportion, compared with the mild ROP group (all *P* < 0.05) (Table 5).

**Table 5:** Comparison of early oxygen parameters between different ROP severity groups (*n* = 107).

Early oxygen parameter	Mild group (*n* = 72)	Severe group (*n* = 35)	*t/χ* ^2^	*P*
Cumulative oxygen duration (h)	153.69 ± 18.46	162.77 ± 13.87	2.838	0.006
Mean FiO_2_ (%)	38.24 ± 7.77	42.97 ± 5.90	3.184	0.002
Maximum FiO_2_ (%)	69.01 ± 15.34	79.60 ± 11.70	3.602	<0.001
SpO_2_ target range time proportion (%)	72.24 ± 12.10	63.83 ± 9.56	3.599	<0.001
Intermittent hypoxia frequency (episodes/d)	11.03 ± 4.27	16.83 ± 6.75	4.650	<0.001

ROP, retinopathy of prematurity; FiO_2_, fraction of inspired oxygen; SpO_2_, pulse oxygen saturation.

### 3.6 Multivariable logistic regression analysis of adjusted associations between early oxygen parameters and severe ROP

Among the 107 infants with ROP, 35 were classified as severe ROP and 72 as mild ROP. Multivariable logistic regression analyses were performed to evaluate whether early oxygen-related parameters remained associated with severe ROP after adjustment for neonatal maturity-related factors. Gestational age, birth weight, and surfactant use were included as adjustment variables, and each oxygen parameter was entered separately into the regression models to minimize potential multicollinearity.

After adjustment, none of the evaluated oxygen-related parameters showed statistically significant associations with severe ROP (all *P* > 0.05). Specifically, cumulative oxygen duration (OR = 0.993, 95% CI: 0.971–1.015, *P* = 0.511), mean FiO_2_ (OR = 0.982, 95% CI: 0.893–1.080, *P* = 0.708), maximum FiO_2_ (OR = 0.994, 95% CI: 0.958–1.031, *P* = 0.755), SpO_2_ target range time proportion (OR = 1.004, 95% CI: 0.952–1.058, *P* = 0.885), and intermittent hypoxia episode frequency (OR = 1.082, 95% CI: 0.880–1.331, *P* = 0.453) were not significantly associated with severe ROP after adjustment. Variance inflation factor analysis indicated no significant multicollinearity among variables included in the models (all VIF<5) (Table 6).

**Table 6:** Multivariable logistic regression analysis of early oxygen parameters associated with severe ROP among infants with ROP.

Variable	OR	95% CI	*P* value
Model 1: Cumulative oxygen duration			
Gestational age	0.621	0.279–1.383	0.243
Birth weight	2.655	0.194–36.324	0.464
PS use	0.651	0.251–1.692	0.379
Cumulative oxygen duration	0.993	0.971–1.015	0.511
Model 2: Mean FiO_2_			
Gestational age	0.628	0.281–1.400	0.255
Birth weight	2.656	0.195–36.168	0.463
PS use	0.654	0.252–1.697	0.383
Mean FiO_2_	0.982	0.893–1.080	0.708
Model 3: Maximum FiO_2_			
Gestational age	0.627	0.281–1.400	0.255
Birth weight	2.662	0.195–36.280	0.463
PS use	0.654	0.252–1.699	0.383
Maximum FiO_2_	0.994	0.958–1.031	0.755
Model 4: SpO_2_ target range time proportion			
Gestational age	0.618	0.277–1.381	0.241
Birth weight	2.672	0.196–36.422	0.461
PS use	0.643	0.248–1.666	0.363
SpO_2_ target range time proportion	1.004	0.952–1.058	0.885
Model 5: Intermittent hypoxia frequency			
Gestational age	0.638	0.283–1.438	0.279
Birth weight	2.618	0.187–36.585	0.474
PS use	0.636	0.244–1.652	0.352
Intermittent hypoxia frequency	1.082	0.880–1.331	0.453

### 3.7 Correlation between early oxygen parameters and ROP stage

**Figure 1:**
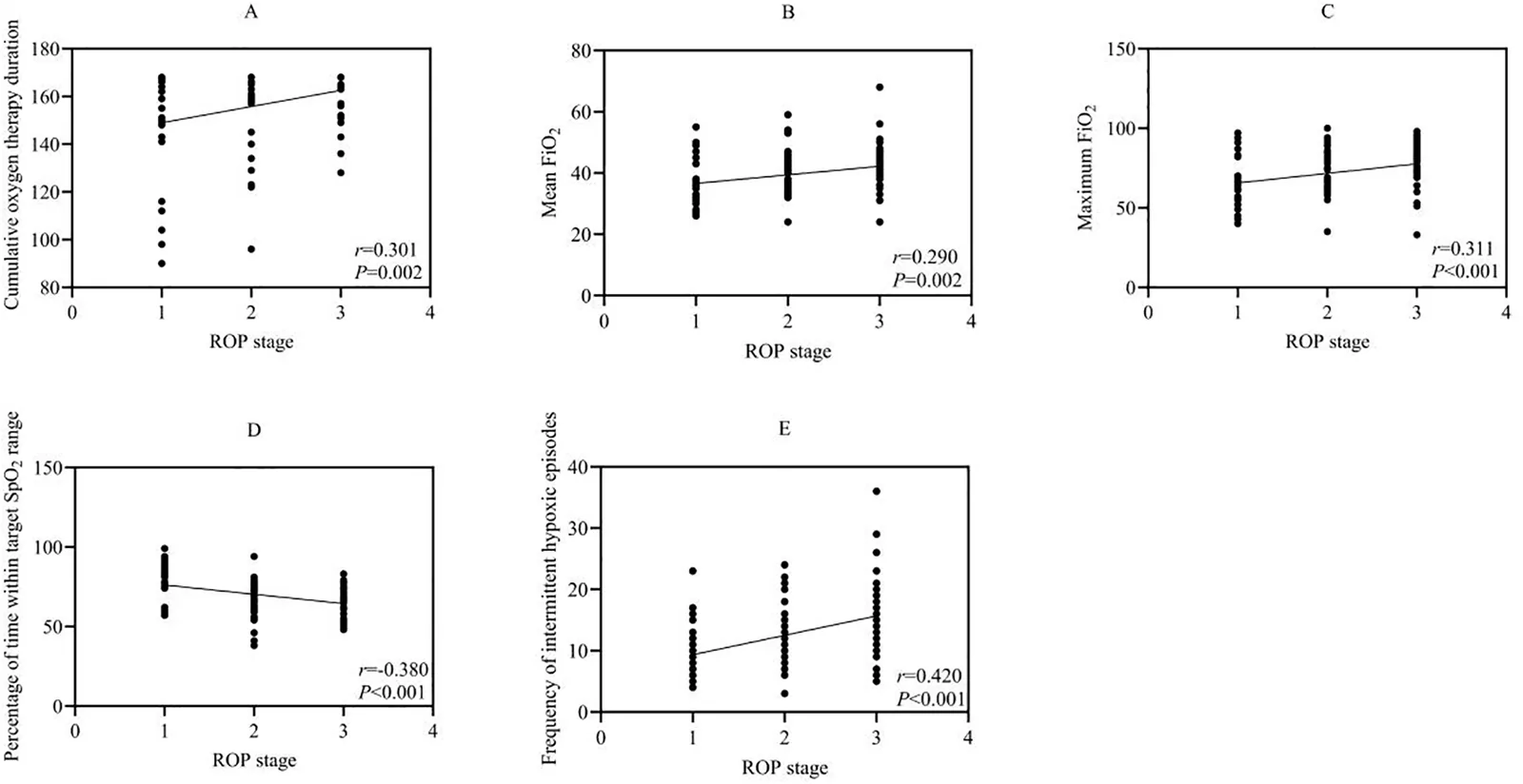
Scatter plots of correlations between each oxygen-related parameter and retinopathy of prematurity (ROP) stage. **(A)** Cumulative oxygen therapy duration vs. ROP stage; **(B)** Mean fraction of inspired oxygen (FiO₂) vs. ROP stage; **(C)** Maximum fraction of inspired oxygen (FiO₂) vs. ROP stage; **(D)** Percentage of time within the target oxygen saturation (SpO₂) range vs. ROP stage; **(E)** Frequency of intermittent hypoxic episodes vs. ROP stage.

Spearman correlation analysis showed that early oxygen-related parameters were significantly associated with ROP stage. Cumulative oxygen duration, mean FiO_2_, maximum FiO_2_, and intermittent hypoxia episode frequency showed positive correlations with ROP stage, whereas SpO_2_ target range time proportion showed a negative correlation (*r* = 0.301, 0.290, 0.311, −0.380, and 0.420, respectively; all *P* < 0.001) (Figure 1).

### 3.8 Discriminative performance of early oxygen parameters for severe ROP among infants with ROP

ROC curve analyses were performed among the 107 infants with ROP to evaluate the ability of early oxygen-related parameters to discriminate severe ROP from mild ROP.

**Figure 2:**
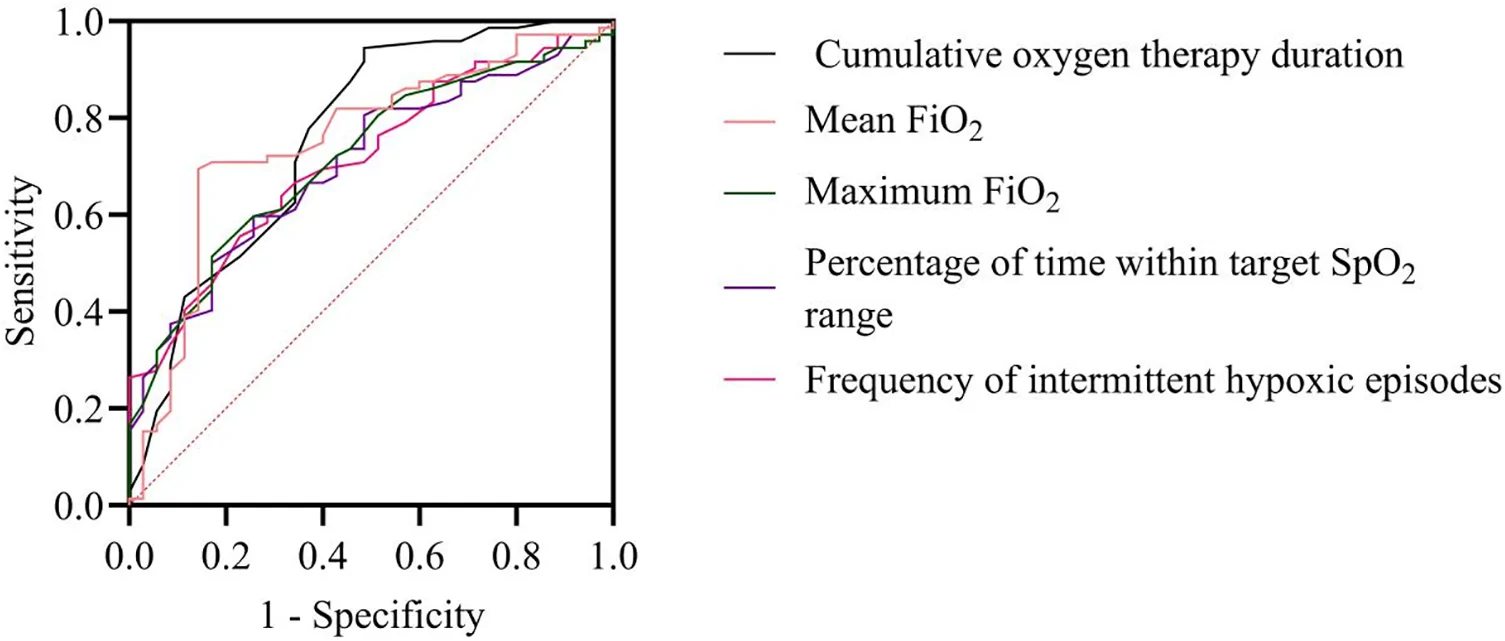
ROC curves of oxygen parameters for discriminating severe from mild ROP among infants with ROP. ROC, receiver operating characteristic; ROP, retinopathy of prematurity; FiO_2_, fraction of inspired oxygen; SpO_2_, pulse oxygen saturation.

The AUC values (95% CI) were 0.728 (0.624–0.831) for cumulative oxygen duration, 0.716 (0.617–0.815) for mean FiO_2_, 0.705 (0.606–0.805) for maximum FiO_2_, 0.712 (0.614–0.811) for SpO_2_ target range time proportion, and 0.761 (0.658–0.863) for intermittent hypoxia episode frequency, indicating moderate discriminative performance (Table 7, Figure 2). No severe ROP cases occurred in the 32–<37 weeks group. Therefore, gestational age-specific ROC analysis was not performed in this subgroup because discrimination analysis was not feasible.

**Table 7:** Discriminative performance of oxygen parameters for severe ROP.

Parameter	AUC (95% CI)	Youden index	Sensitivity	Specificity	*P*
Cumulative oxygen duration	0.728 (0.624–0.831)	0.451	0.743	0.708	<0.001
Mean FiO_2_	0.716 (0.617∼0.815)	0.343	0.829	0.514	<0.001
Maximum FiO_2_	0.705 (0.606∼0.805)	0.340	0.743	0.597	0.001
SpO_2_ target range time proportion	0.712 (0.614∼0.811)	0.327	0.771	0.556	<0.001
Intermittent hypoxia frequency	0.761 (0.658∼0.863)	0.458	0.514	0.944	<0.001

ROP, retinopathy of prematurity; FiO_2_, fraction of inspired oxygen; SpO_2_, pulse oxygen saturation; AUC, area under the curve; CI, confidence interval.

### 3.9 Incremental discriminative value of early oxygen parameters beyond gestational age and birth weight

To evaluate whether oxygen-related parameters provided additional discriminative information beyond conventional maturity-related factors, baseline models incorporating gestational age and birth weight were compared with models additionally including oxygen parameters.

Among infants with ROP, the baseline model incorporating gestational age and birth weight showed limited discrimination for severe ROP (AUC = 0.542, 95% CI: 0.501–0.681). Adding individual oxygen-related parameters did not improve discrimination, and the combined oxygen model showed only a modest increase in AUC (0.587, 95% CI: 0.583–0.766) (Table 8).

**Table 8:** Incremental discriminative value of early oxygen parameters beyond gestational age and birth weight for severe ROP.

Model	Variables included	AUC (95% CI)
Model 1	Gestational age + Birth weight	0.542 (0.501–0.681)
Model 2	Model 1 + cumulative oxygen duration	0.539 (0.521–0.699)
Model 3	Model 1 + intermittent hypoxia frequency	0.532 (0.515–0.696)
Model 4	Model 1 + cumulative oxygen duration + mean FiO_2_ + maximum FiO_2_ + SpO_2_ target range proportion + intermittent hypoxia frequency	0.587 (0.583–0.766)

## 4 Discussion

The results of this study showed that among preterm infants with RDS, the overall ROP incidence was 12.97% and increased significantly with decreasing gestational age, with an ROP incidence as high as 59.57% in the 24–<28 weeks group, markedly higher than that in the 28–<32 weeks and 32–<37 weeks groups. This finding suggests that gestational age remains one of the most core fundamental risk factors for ROP development. The smaller the gestational age, the more immature the retinal vascular development, and the more susceptible the infant is to fluctuations in oxygen exposure and oxidative stress after birth, thereby inducing abnormal angiogenesis and ROP progression. Numerous previous studies have confirmed that extremely preterm infants, due to incomplete retinal vascular development, are more sensitive to hyperoxia and oxygen fluctuations (20–23). The present results are consistent with previous reports and further indicate that this risk may be amplified in the setting of RDS.

This study also found that with decreasing gestational age, birth weight and Apgar scores gradually decreased, while PS use rate, oxygen therapy duration, and FiO_2_ levels gradually increased, suggesting that infants with lower gestational age have more severe illness and a higher degree of oxygen dependence. This finding aligns with the pathophysiology of RDS (24). The smaller the gestational age, the greater the deficiency in alveolar number and PS, leading to decreased lung compliance and more pronounced oxygenation impairment, thus requiring higher concentrations and longer durations of oxygen support. However, prolonged high oxygen exposure can induce retinal vasoconstriction, suppression of VEGF expression, and oxidative stress injury, ultimately promoting ROP development (25). Therefore, the increase in ROP is not simply caused by lower gestational age alone but may be the result of a continuous pathological chain of lower gestational age–more severe RDS–higher oxygen exposure.

Among infants with ROP, the present study further revealed that with decreasing gestational age, the severity of ROP stage, incidence of plus disease, and proportion of severe ROP all increased markedly, with the 24–<28 weeks group predominantly having severe ROP. This finding suggests that extremely preterm infants not only have a higher incidence of ROP but are also more likely to progress to severe lesions requiring interventional treatment. This may be related to the prolonged immaturity of retinal vessels in extremely preterm infants, along with a greater susceptibility to oxygen saturation fluctuations and intermittent hypoxic events during oxygen therapy, thereby exacerbating retinal ischemia-reperfusion injury (26). The increased proportion of plus disease also indicates more pronounced abnormal dilation and tortuosity of posterior pole vessels, reflecting enhanced pathological vascular proliferation activity. One of the important findings of this study is that oxygen parameters differed significantly between ROP severity groups. The severe ROP group had significantly higher cumulative oxygen duration, mean FiO_2_, maximum FiO_2_, and intermittent hypoxia episode frequency, and a significantly lower SpO_2_ target range time proportion compared with the mild group. This indicates that ROP risk is associated not only with the total amount of oxygen exposure but also with the stability of oxygen therapy and the degree of oxygen fluctuations. Prolonged oxygen therapy may increase sustained hyperoxia exposure, while frequent hypoxic events may create repeated hypoxia-reoxygenation cycles, generating large amounts of oxygen free radicals and initiating inflammatory cascades, further promoting abnormal vascular proliferation (2).

Spearman correlation analysis showed that cumulative oxygen duration, mean FiO_2_, maximum FiO_2_, and intermittent hypoxia episode frequency were each positively correlated with ROP stage, while SpO_2_ target range time proportion was negatively correlated with ROP stage. Among these, intermittent hypoxia episode frequency showed the highest correlation, suggesting that oxygen fluctuations may be more indicative of ROP progression risk than hyperoxia alone. A growing number of recent studies have suggested that stably maintaining SpO_2_ within the target range is more important than simply avoiding hyperoxia (13, 27, 28). Frequent hypoxic events can lead to abnormal activation of hypoxia-inducible factor (HIF) and VEGF, making pathological neovascularization more pronounced. Our results further support the “oxygen fluctuation injury” theory, indicating that in clinical oxygen therapy, not only sustained hyperoxia should be avoided, but also large fluctuations in SpO_2_ and the occurrence of hypoxic events should be minimized as much as possible. Although several oxygen-related parameters differed significantly between mild and severe ROP groups, these associations were attenuated after adjustment for gestational age, birth weight, and surfactant use. This finding suggests that oxygen exposure may partly reflect underlying immaturity and disease severity rather than acting as an isolated determinant of severe ROP.

ROC curve analysis showed that early oxygen-related parameters had moderate discriminative performance for distinguishing severe from mild ROP among infants who had already developed ROP, with AUC values ranging from 0.705 to 0.761. Among these parameters, intermittent hypoxia episode frequency showed the highest AUC, whereas cumulative oxygen duration demonstrated relatively higher sensitivity. However, these findings should be interpreted cautiously because the discriminative ability of individual oxygen parameters remained limited. Furthermore, additional modeling demonstrated that incorporating oxygen-related parameters provided only minimal improvement beyond gestational age and birth weight, suggesting that oxygen exposure patterns may partly reflect underlying biological immaturity and disease severity rather than providing substantial independent discriminative information. This finding should be interpreted within the context of the analysis population, which included only infants with established ROP. Therefore, gestational age may have stronger relevance for predicting ROP occurrence than for differentiating severity among affected infants. Therefore, oxygen parameters alone are unlikely to be sufficient for clinical decision-making, but may serve as supplementary indicators when combined with established neonatal risk factors and clinical assessment. Future studies integrating dynamic oxygen parameters with comprehensive clinical risk models and validating them in multicenter cohorts are warranted.

This study provides several clinical implications. First, by evaluating multiple dynamic oxygen-related parameters, this study extends previous analyses focusing mainly on oxygen duration or FiO_2_ levels and highlights the potential relevance of oxygen stability characteristics in relation to ROP severity. Second, the gestational age-stratified analysis emphasizes that extremely preterm infants represent a particularly vulnerable population with higher ROP incidence and severity. In clinical practice, careful oxygen management, maintenance of appropriate SpO_2_ targets, and reduction of unnecessary oxygen fluctuations remain important components of neonatal care. However, the present findings do not support modification of current ROP screening protocols or the use of oxygen parameters as independent criteria for intervention. Instead, these parameters may provide additional information for risk characterization within existing screening frameworks, but their clinical utility requires further prospective validation. Recent advances in ROP screening strategies, including pediatrician-performed digital retinal imaging, have demonstrated the potential value of accessible imaging approaches for improving early detection, particularly in settings with limited ophthalmologic resources (29). Prospective multicenter studies are needed to determine whether dynamic oxygen indicators can provide additional clinical benefit when incorporated into established ROP risk assessment strategies.

Nevertheless, this study has several limitations. First, as a single-center retrospective study, selection bias and information bias may exist. Second, this study mainly analyzed oxygen parameters during the first 7 d after birth, whereas ROP is a long-term progressive disease, and subsequent oxygen exposure and mechanical ventilation strategies may also affect ROP outcomes. Third, this study did not further include potential confounding factors such as sepsis, blood transfusion, or patent ductus arteriosus for multivariate adjustment, and thus further refinement is needed. Other neonatal complications, including sepsis, patent ductus arteriosus (PDA), and bronchopulmonary dysplasia (BPD), were not included in the final model because of the limited number of severe ROP events, and their potential effects should be further evaluated in larger prospective cohorts. In addition, although this study shows associations between oxygen parameters and ROP stage, it does not fully establish causality, and future multicenter, large-scale prospective studies are needed for further validation.

In summary, this study demonstrates that among preterm infants with RDS, both the incidence and severity of ROP increase substantially with decreasing gestational age. Early oxygen-related parameters, including cumulative oxygen duration, FiO_2_ exposure, SpO_2_ target range time proportion, and intermittent hypoxia frequency, were associated with ROP stage and showed moderate discriminative performance for distinguishing severe from mild ROP among affected infants. However, these parameters provided limited additional discriminative value beyond gestational age and birth weight. Therefore, dynamic oxygen indicators may represent candidate markers for risk characterization, but their clinical utility requires further validation in prospective multicenter studies.

## Data Availability

The original contributions presented in the study are included in the article/Supplementary Material, further inquiries can be directed to the corresponding author.
